# A Blockchain Implementation Prototype for the Electronic Open Source Traceability of Wood along the Whole Supply Chain

**DOI:** 10.3390/s18093133

**Published:** 2018-09-17

**Authors:** Simone Figorilli, Francesca Antonucci, Corrado Costa, Federico Pallottino, Luciano Raso, Marco Castiglione, Edoardo Pinci, Davide Del Vecchio, Giacomo Colle, Andrea Rosario Proto, Giulio Sperandio, Paolo Menesatti

**Affiliations:** 1 Consiglio per la Ricerca in Agricoltura e l’Analisi dell’Economia Agraria (CREA)—Centro di Ricerca Ingegneria e Trasformazioni Agroalimentari, Via della Pascolare 16, 00015 Monterotondo, Italy; simone.figorilli@crea.gov.it (S.F.); francesca.antonucci@crea.gov.it (F.A.); federico.pallottino@crea.gov.it (F.P.); giulio.sperandio@crea.gov.it (G.S.); paolo.menesatti@crea.gov.it (P.M.); 2 Microsoft S.r.l., Viale Pasubio 21, 20154 Milano, Italy; luraso@microsoft.com (L.R.); marco.castiglione@microsoft.com (M.C.); epinci@microsoft.com (E.P.); davide.delvecchio@microsoft.com (D.D.V.); 3 Effetreseizero Srl, Spinoff CREA, Via dei Solteri 37/1, 38121 Trento, Italy; giacomo.colle@f360.it; 4 Department of AGRARIA, Mediterranean University of Reggio Calabria, Feo di Vito, 89122 Reggio Calabria, Italy; andrea.proto@unirc.it

**Keywords:** IoT, sensors, infotracing, RFID, ARDUINO^®^

## Abstract

This is the first work to introduce the use of blockchain technology for the electronic traceability of wood from standing tree to final user. Infotracing integrates the information related to the product quality with those related to the traceability [physical and digital documents (Radio Frequency IDentification—RFID—architecture)] within an online information system whose steps (transactions) can be made safe to evidence of alteration through the blockchain. This is a decentralized and distributed ledger that keeps records of digital transactions in such a way that makes them accessible and visible to multiple participants in a network while keeping them secure without the need of a centralized certification organism. This work implements a blockchain architecture within the wood chain electronic traceability. The infotracing system is based on RFID sensors and open source technology. The entire forest wood supply chain was simulated from standing trees to the final product passing through tree cutting and sawmill process. Different kinds of Internet of Things (IoT) open source devices and tags were used, and a specific app aiming the forest operations was engineered to collect and store in a centralized database information (e.g., species, date, position, dendrometric and commercial information).

## 1. Introduction

The capacity to track the complete product supply chain in both industries and agriculture is nowadays possible with the implementation of automated identification systems establishing a link among the product and a database of the product and processes it undergoes [1]. An infotracing system is a process to keep records revealing the trail of an input from suppliers to customers [2]. This aspect is receiving an increasing interest in the forestry sector, [3,4,5,6]. Generally, a traceability system relies on the capacity to track product one step forward and one step back at any point in the supply chain, eventually allowing the quick and effective implementation of corrective actions where and when needed. Traceability is important for all businesses, including importers and retailers, to be able to trace products [1,7].

In Europe, two certification systems for sustainable forest management have been developed (i.e., the Forest Stewardship Council (FSC) and Programme for the Endorsement of Forest Certification (PEFC) schemes) [8]. The European Regulation 995/2010 (EUTR) or Timber Regulation prohibits the use and trading of products of illegal origin and obliges the adoption of an internal system of due diligence for the subjects who place wood products and derivatives on the European market, with the need to certify the product origin and to trace the flows [5]. Sperandio et al. [8] remarked on the importance of the use of traceability systems within the wood workflow. In details, the work specifies the necessity to trace information from the standing tree through the whole chain as a necessary condition to certify the origin of the material. This represents the documented correlation between raw wood and original tree populations with the storage of all data linked to the origin and the authorization procedure of the cut. In such a context, infotracing systems can provide a reference web interface to access the product info card displaying all information and data released as feedback by the manufacturer, wholesaler, reseller, retailer and consumer [2].

Generally, one persistent problem in several supply chains is the non-optimal use of resources that can be solved by introducing automated and electronic real-time traceability solutions for the entire working flow [9]. In all timber producing countries, rules and regulations require specific markings that must be placed at the ends of all legal logs. The identification and marking of suitable trees for felling represent the main operations that the forest technician must perform as a technical and administrative obligation, on which the authorization procedure for cutting trees is based. The timber marking represents numbers and letters of identification pressed on the wood surface. The application of Radio Frequency IDentification (RFID) technology, starting from this phase, could be a valuable tool for a possible optimization of this operation in terms of time, materials and costs along the entire supply chain, from the time of the timber marking, to the subsequent traceability of the wood product up to its final destination. As an electronic technology, RFID technology reduces some of the information gaps, especially in logistics, enabling a real-time visibility into supply chains [10]. Generally, the production logistics processes can occupy up to 95% of the execution time of the whole production process, significantly influencing the overall production efficiency [11]. RFID technology can achieve automatic traceability by enabling us to connect the physical world objects with their virtual counterparts. This traceability system needs to use a temporal data model to support tracking of the raw material and monitoring of the processes [8]. Picchi et al. [6] evaluated the performance of two tag models and two fixing options thorough the timber supply chain in steep terrain during cable yarding and logistic operations. Björk [4] claims that an economic benefit can be achieved along the supply chain even with partial tagging of the whole load of logs, which still allows for a certain degree of traceability of the loads. Kaul [12] tested the performance of several RFID tag models both in bulk reading (e.g., a full truckload of tagged logs) and single items identification.

Infotracing integrates the information related to the quality of the product with those related to the traceability [physical and digital documents (RFID architecture)] within an online information system whose steps (transactions) can be made safe to evidence of alteration through the blockchain. The blockchain is a decentralized and distributed ledger that keeps records of digital transactions in such a way that makes them accessible and visible to multiple participants in a network while keeping them secure without the need of a centralized certification organism. As a blockchain is a decentralized database, no one regulates it or owns it, and once the data uploaded to the blockchain, it becomes immutable, not allowing data to be tampered with or falsified [13]. The blockchain consists in a linear sequence of small encrypted datasets called ‘blocks’, which contain timestamped batches of transactions [14]. As reported by Lin et al. [15], each of these blocks contains a reference to its precedent block and an answer to a complex mathematical puzzle, which serves to validate the transactions it contains as a decentralized ledger system of transaction records, which is distributed across a network of computers or databases.

This paper reports the implementation of a blockchain architecture within the wood chain electronic traceability. The infotracing system is based on RFID open source technology underlining the difference with the traditional documental/mechanical methods. The entire forest wood supply chain was simulated in the Calabria Region in Southern Italy, from standing trees to the final product passing through tree cutting (felling, harvesting, processing) and sawmill process. Recent studies have explored the role of the Internet of Things (IoT) as an enabler of real-time quality management and control instruments in the supply chain [16]. Blockchain could help in achieving major security requirements in IoT [17]. Different kinds of IoT open source devices and tags were used, and a specific app aiming the forest operations was developed to collect and store in a centralized database information such as species, date, position, number of logs produced for each tree, dendrometric and commercial information.

## 2. Materials and Methods

### 2.1. Wood Traceability Phases

Ten trees were followed using RFID technology along their supply/processing chain from the forest stand to the sawmill plant in Southern Italy, near the municipality of Cardinale (Province of Catanzaro).

**Figure 1:**
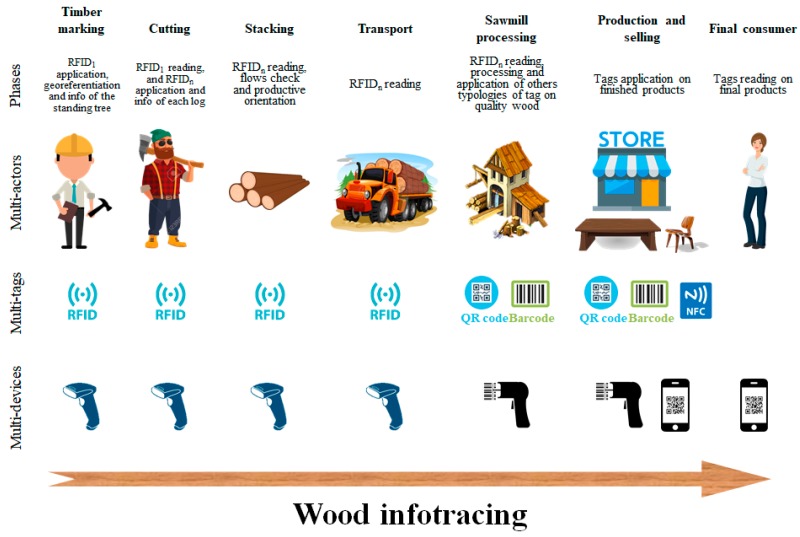
Wood infotracing phases and relative multi-actors, multi-tags and multi-devices: in the timber marking phase the application of the first RFID (RFID1) above the cut and its first tag association with information on the database was achieved; in the cutting phase, additional RFID (RFIDn) tags (one for each derived log; the same used for the Timber marking), was applied on the cutting portion for each log (excluding branches and pieces of lesser quality); in the stacking phase the RFIDn was read to check flows and to orient the production; in the transport phase the RFIDn was read during the logs transport; in the sawmill processing phase each log was recognized by an antenna and the RFIDn information was associated to the database. In this phase the most quality pieces were marked with QR code stickers, to enhance and certify the timber and its origin; in the production and selling phase the tags were applicated on the final products; in the final consumer phase the tags were read on final products.

The wood infotracing phases (Figure 1) are shown below:

- Timber marking: application of the first RFID (RFID1) of the Class 1 Gen 2 (coin shaped with central hole) above the cut at the moment of the tree identification. This first tag associates the information on the database of the standing tree: tree marking date, tree GPS point, species, diameter at breast height, qualitative class, other information;
- Cutting: additional RFID (RFIDn) tags (one for each derived log; the same used for the timber marking), were applied on the cutting portion for each log (excluding branches and pieces of lesser quality). Each RFID is uniquely associated with the tree, thus preserves the association with the RFID information applied in the first phase, and adds the following data related to each single log: cutting date, log length, log average diameter, wood quality categories, other information;
- Stacking: RFID2 reading to check flows and to orient the production;
- Transport: RFID2 reading during the logs transport;
- Sawmill processing: each log is recognized by an antenna and the RFID2 information is associated to the database: date of entry into the sawmill; description and number of parts in which the log is decomposed; additional qualitative information; other information. The pieces of the best quality are then marked with QR code stickers, to enhance and certify the timber and its origin.
- Production and selling: tag application on the final products;
- Final consumer: Tag reading on the final product.

### 2.2. RFID Technologies

The open source prototype system based on RFID technology has been implemented using different wireless communication protocols for each phase of the infotracing flow. Through the RFID antennas the tags are read in the various phases and, in particular in an outdoor context, through a portable reader, sent the codes to a smartphone via Bluetooth that, with a customized app (SmartTree, described below), stores the code and the related additional information entered by the operator.

The customized portable reader was developed using open sources technologies consisting in an ARDUINO^®^ Micro Controller Unit (MCU) with integrated Bluetooth (Bluno Mega 2560) and a RFID antenna interfaced through serial communication. The communication protocol with the antenna was implemented by integrating cyclic redundancy control (CRC) to reduce errors in reading. Also, a serial communication was implemented for the transmission via Bluetooth with a smartphone.

In the sawmill a long-range UHF RFID antenna was used (RFID102 UHF Reader; Go Young International Ltd., Shanghai, China; it supports ISO18000-6B, ISO18000-6C (EPC C1G2) protocol tags). The reading and management software of this antenna was developed using the Software Development Kit (SDK) released by the producer.

**Figure 2:**
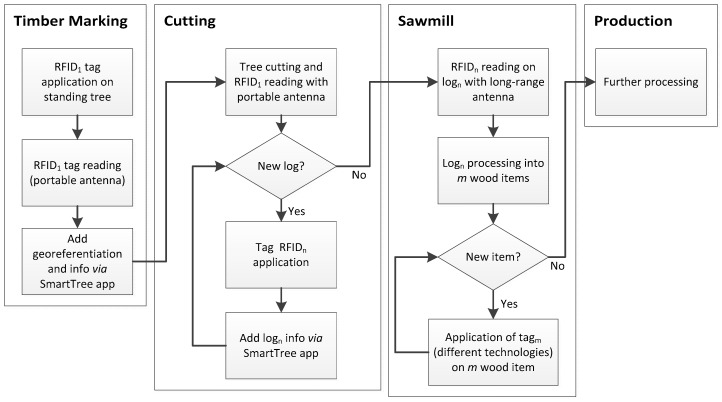
Diagram of the phases in relation to the RFID technologies. In particular of the “timber marking”, “cutting”, “sawmill” and “production” phases.

The RFID standard used is UHF at 860 MHz. The tags used were Class 1 Gen 2, that, compared to the predecessors Class 1 Gen 1, did not contain any kind of support for the processed data security. Class 1 Gen 2 tags support two systems useful for the processed data security such as the TID code (the serial tag ID) and the access/kill passwords. Three kinds of different tags (Go Young International Ltd.) have been tested: one nail shaped (head size Ø 2 cm) and two centrally pierced coin shaped ones (diameters: 3 cm and 4 cm respectively). For these tags, the mean higher measurement distance from both the long-range and the portable antenna was calculated for each RFID tag (ten replicates each). Figure 2 presents a diagram of the phases in relation to the RFID technologies developed in this study.

### 2.3. Blockchain Architecture

To readily leverage the blockchain technology was used the Azure Blockchain Workbench (Microsoft Corporation, Redmond, WA, USA) with a cloud deployment. Azure Blockchain Workbench is a collection of Azure services and capabilities designed to help create and deploy blockchain applications to share business processes and data with other organizations. Azure Blockchain Workbench provides the infrastructure scaffolding for building blockchain applications enabling developers to focus on creating business logic and smart contracts. It also simplifies the creation of blockchain applications by integrating several Azure services and capabilities to help automate common development tasks.

The Blockchain Workbench exposes REST APIs and message-based APIs that can be used to integrate data with existing systems. Blockchain Workbench can transform messages sent to its message-based API to build transactions in a format expected by that blockchain’s native API. Workbench can sign and route transactions to the appropriate blockchain.

Azure Blockchain Workbench also facilitates the analysis of blockchain events and data by automatically synchronizing data on the blockchain to off-chain storage. Instead of the resource consuming task of extracting data directly from the blockchain, it is possible to query an off-chain database system such as SQL Server.

Development of application inside the workbench is done by writing a JSON configuration file and a related smart contract code to describe state machines can be built to represent the application logic. Currently the workbench supports Ethereum smart contracts written in the Solidity language.

Among the services deployed by the Microsoft Azure Blockchain Workbench there’s also a native support to integrate IoT devices directly using an Azure IoT Hub. Such a service allows a secure integration of IoT devices directly to the developed application.

**Figure 3:**
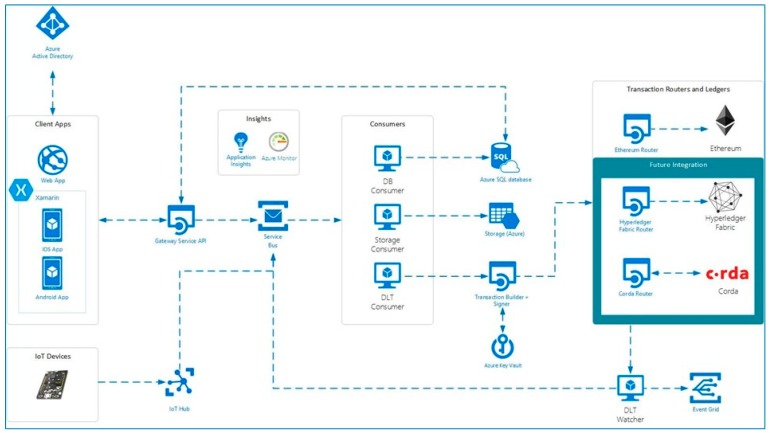
Azure workbench blockchain flowchart: list of activated services on Azure cloud and their connections.

Figure 3 depicts the current state of the art of the services deployed by the current Microsoft Azure Blockchain Workbench layout. It also shows not only the integration of the components starting with the Internet-facing Gateway Services API and IoT Hub, but also the extensible nature of the framework that allows further expansion by plugging in custom transaction consumers and possible future integration with different ledger technologies.

**Figure 4:**
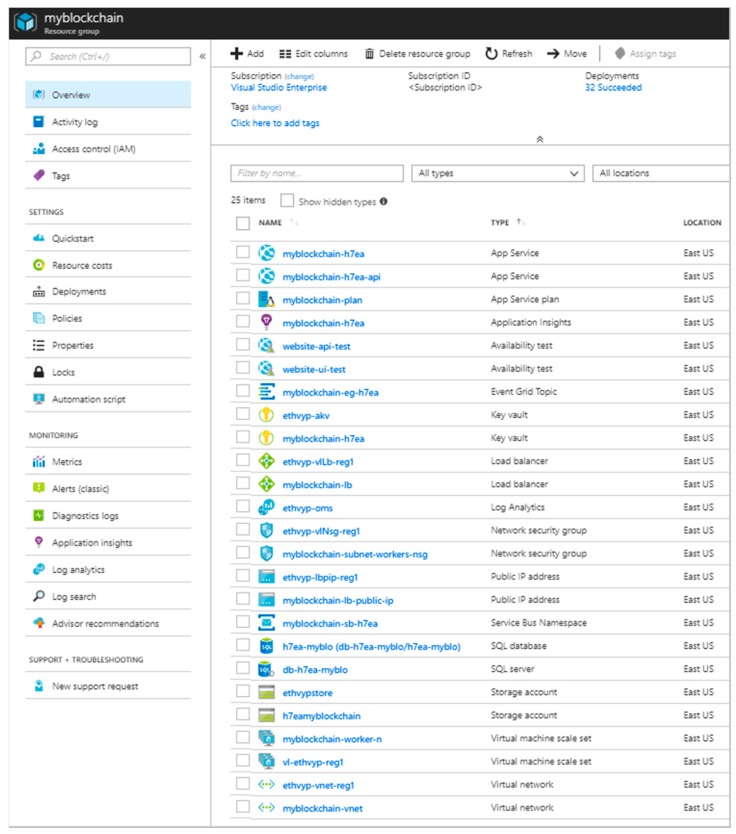
Screenshot of the fully populated resulting resource group of the Azure workbench blockchain.

The screenshot of the resource group of the Azure workbench blockchain is shown in Figure 4.

A full workbench deployment includes:

- One Event Grid Topic.
- One Service Bus Namespace.
- One Application Insights.
- One SQL Database (Standard S0).
- Two App Services (Standard).
- Two Azure Key Vaults.
- Two Azure Storage accounts (Standard Learning Record Store—LRS).
- Two Virtual machine scale sets (for validator and worker nodes).
- Two Virtual Networks (including load balancer, network security group, and public IP address for each virtual network).
- Optional: Azure Monitor.

### 2.4. Blockchain Interconnection

**Figure 5:**
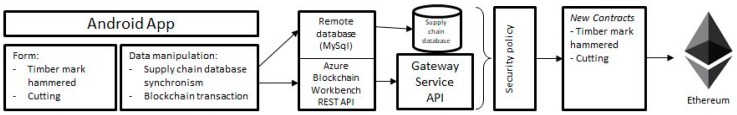
Interconnection diagram between the app developed (SmartTree, described below) and the blockchain.

The pre-existing software architecture has been modified to interact with the blockchain one. The changes concerned the synchronization part with the central server (the supply chains’ database) adding a data manipulation software layer to make it compatible with the relative contract present in the blockchain. In addition to the data manipulation, a connection section to the Gateway service Application Programming Interface (API, Figure 3) has been added to communicate with the Azure blockchain workbench. Through the ‘security policy’ element (Figure 5), it is possible to implement parallel mechanisms of consolidation and coherence of information before the permanent validation and storage in the blockchain (Figure 5). In relation to the information that was managed via app, the related contracts (timber marking and cutting) were created with the contract status management (Figure 5).

### 2.5. App (SmartTree) for In-Field Data Collection

An app (SmartTree) for the wood traceability data collection has been developed to support the in-field operation from the timber marking to cutting phases, providing operators with a simple and easy-to-use tool for smartphones. The information registered through the app is stored on an internal database and subsequently synchronized on a remote server. In addition, the app uses some devices inside the smartphone (i.e., GPS, Bluetooth) to collect data. The GPS receiver was used to collect the trees geographical position.

The connection between the smartphone with the external customized portable RFID reader was realized via Bluetooth (specified in Section 2.2).

Internet connectivity was needed for the synchronization on the remote server; this operation could be achieved both in-field (if the internet connection is available) or after field operations, when the internet signal become available.

**Figure 6:**
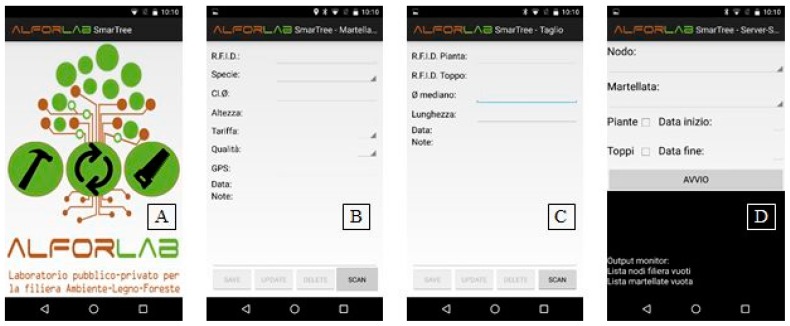
(**A**) Main activity of the SmartTree app (written in Italian to be used by local operators); (**B**) Activity of the timber marking form [Species (Specie), Diametric class (Cl. Ø), Height (Altezza), Tariffs (Tariffa; from 1 to 9), Quality (Qualità; A, B, C, D), GPS (automated inserted and unmodifiable), Date (Data; automated inserted and unmodifiable), Note]; (**C**) Activity of the cutting form [Median Diameter (Ø mediano), Length (Lunghezza), Date (Data; automated inserted and unmodifiable), Note]; (**D**) Activity of the synchronization phase with the remote server and blockchain. The app was developed to be used by Italian users [Node (Nodo), Timber marking (martellata), Ttree (Piante), Logs (Toppi), Start date (Data inizio); End date (Data fine)].

The app (Figure 6A) was structured to operate during the timber marking phase or the cutting and remote synchronization phase.

Each activity of the SmartTree app was described below and represented in Figure 6:

- Main activity: the start page allows to select the phase to be executed (Timber marking or Cutting or Remote synchronization);
- Timber marking phase (Figure 6B): through the “scan” button, the Bluetooth connection of the smartphone to the customized portable RFID reader is linked. The device is in a waiting state ready to receive the tag RFID1 unique code that identifies the tree. When the RFID code is received, the app checks for the presence of the code. If it is an existing code, all data will be loaded and could be modified up to 2 h.; while, if it is a new code, the fields will be activated to enter the following information: Species (Specie), Diametric class (Cl. Ø), Height (Altezza), Tariffs (Tariffa; from 1 to 9), Quality (Qualità; A, B, C, D), GPS (automated inserted and unmodifiable), Date (Data; automatically inserted and unmodifiable), Note: once inserted, the data will be saved on an internal database. Data updating, and deletion functions have also been implemented and are available for up to 2 h;
- Cutting (Figure 6C): as reported above, the scan button allows the reading of a RFID tag via Bluetooth. The procedure consists in: i. reading the RFID1 positioned on the standing tree to be cut, ii. reading and insert the RFIDn tags on each log deriving from the cut tree, iii. filling the following information for each log: Median Diameter (Ø mediano), Length (Lunghezza), Date (Data; automated inserted and unmodifiable), Note: control procedures to verify the data consistence and coherence has been implemented. As above described, data updating, and deletion functions have also been implemented and are available for up to 2 h;
- Remote synchronization (Figure 6D): this section manages the synchronization with the remote server and the blockchain. It is activated into two steps: the first, before the in-field operation (timber marking or cutting), allows one to proceed with the operation once the activation code provided by the authoritative institution has been inserted, the second to send the information acquired during (if internet connection is available) or at the end of the specific in-field operation, specifying the temporal interval and the type of data (i.e., tree or log). In the first step the Azure Blockchain workbench login took place (Figure 3). This releases a token to authorize the app to operate in the Azure services. In the second step the data was adjusted in the form of the relative blockchain contract for sending it to the API service gateway and then, for writing it within the blockchain (Figure 3). During synchronization, the app checks to prevent the insertion of duplicate elements on the server and the insertion of unauthorized plants and logs. Another control is carried out on the data coherence, exploiting the blockchain and the supply chain database, through the control policies defined during the integration phase. All synchronization phases are displayed on the screen to get feedback progress.

## 3. Results

On 16 May 2017, the 10 chestnut standing trees were timber marked. Each standing tree was cut, getting 48 logs on which the tags were placed on 26 May 2017. Once the logs were stacked, they were transported to the sawmill on 29 May 2017 and processed in the same date. The quality timbers were tagged with QR codes.

The mean higher measurement distances (based on ten replicates) calculated from both the long-range and the portable antenna for both coin and nail shaped tags, are reported in Table 1. For this analysis the circular shaped tag of 4 cm was chosen.

**Figure 7:**
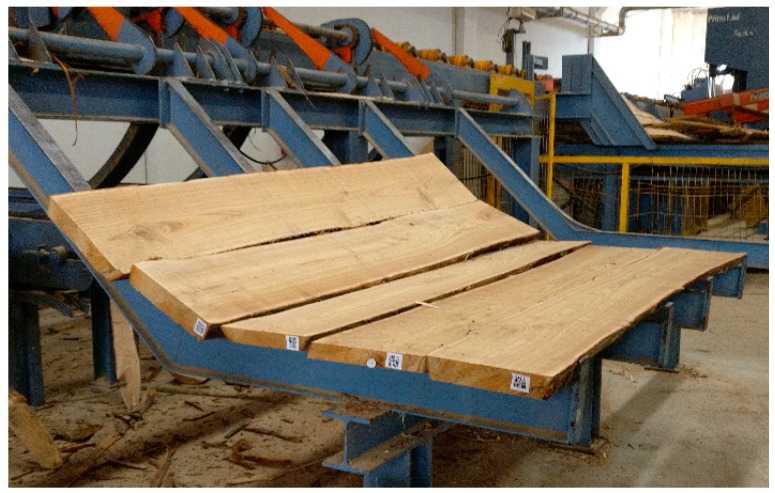
Chestnut boards obtained and marked with QR code at the sawmill.

In details, a chestnut tree near Cardinale (CZ, South Italy, 38°38′32.59″ North, 16°24′12.34″ East) has been tagged with RFID1 inserting all the information through the app. Then, the tree was cut and on each of its five logs of 4.2 m, other tags (RFIDn) have been inserted which, always through the app, store the initial information and add other relative information to the size of each log. Once the logs were equipped, they were transported to the sawmill where they were processed. From one of these logs, boards were obtained, marked with QR code to maintain the previous information (Figure 7), and used to create a table.

**Figure 8:**
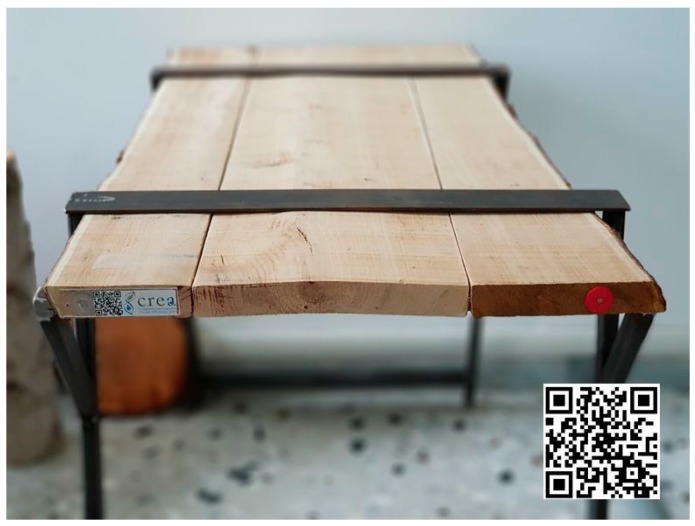
Table made with marked chestnut boards. It is possible to observe the RFID devices (red on the right), QR code (on the left of the word CREA and on the right of the image) and NFC (on the left of the QR code). The QR code shows the web page that the consumer can consult to view the information related to the boards with which the table was made.

The final product (a table; Figure 8) has been tagged with RFID, QR code and Near Field Communication (NFC) referring to a web page that the final consumer can consult through their smartphone (http://www.crea-it.it/nfc/alforlab.html).

The deployment of an Azure Blockchain Workbench is a relatively straight forward process: given an existing tenant (directory) and subscription, there are a number of steps to follow detailed in the documentation web site (https://docs.microsoft.com/en-us/azure/blockchain-workbench/blockchain-workbench-deploy) that mainly revolve around a wizard-driven solution template that guides the configuration and ultimately deploys an Azure Resource Manager template with all the required resources.

## 4. Discussion

### 4.1. Implications for Theory

This is the first work which introduce the blockchain technology for the electronic traceability of wood from standing tree to final user. There are many different aspects that should be discussed considering the advantages in the introduction of an electronic traceability on the chain-of-custody of wood products [8]. First of all the possibility of global georeferenced real-time monitoring, conducted by forestry administrators, on the operations status such as the assignment of lumber to be cut and the information flow of the resulting operations to be conducted. Other important aspects that could benefit from an early timber evaluation regards the possibility (before administrative auction operations) of precisely defining areas of interest and quantities to be cut (including size and species) of the lots that will arrive on the market. Moreover, the system produce the immediate possibility to detect and underline the presence (even a minority or singular tree) of any material of particular value (e.g., in terms of size/quality and/or species). Furthermore, the electronic wood traceability, with the introduction of blockchain, gives the opportunity to extract “backward inferences” from products endowed with a concrete single tree traceability in each processing phase, in terms of the correlation of performance data and technological quality found after the first processing, with the geo (topo) graphic origin, even with the possibility of the backward improvement of the dendro-auxometric estimation adopted in planning [5].

It is then very clear that the introduction of such systems would lead to strong contrasts to illegal cutting [18,19] at least regarding the most valuable timber. Indeed, this would be possible where the assortments from locally processed sawmill are controllable for the mandatory presence of tags that report to centralized unmodifiable (blockchain) archives which also contain georeferenced data and the relative cutting authorization.

### 4.2. Implications for Practice

Blockchain technology, certainly, being based on a decentralized database could efficiently manage transactions. The blockchain is shared between all nodes of the network. The same information is present in all nodes and therefore becomes unmodifiable. In any case, it will not change the history of these same information. Therefore, this technology introduces a new level of transparency and efficiency, allowing the network to achieve and create confident transactions in an untrustworthy environment [20].

Paletto and Notaro [21] evidenced how about the 30% of a pool of wood manufacturers interviewed in Italy were willing to pay a mean premium price of 2.40% for certified wooden planks, while, 19.0% of them are willing to pay a premium of 2.68% for certified wooden panels. Sperandio et al. [8] conducted an economic evaluation of RFID and open source technologies implementation from the forest to the industry as a real possibility to streamline operations and to use resources more efficiently. This suggests how the use of such technologies for wood traceability is already feasible and economically sustainable, being most of the proposed scenarios compatible with an increasing price of traced wood lower than 5% and so compatible with the above mentioned results. The cost increases ascribable to the blockchain implementation, depending from the supply chain dimension, could be considered economically sustainable too, as a rising technology. With the rapid development of IT, this application cost will rapidly decrease [22].

### 4.3. Limitations and Further Research Directions

Moreover, the potential future implementation of bitcoin in the blockchain of wood products will stimulate all the actors of the supply chain, from forestry operators to consumers [23] to use, sustain and chose blockchain certified wood products.

## 5. Conclusions

Overall, blockchain as a technology has the potential to change the way how traceability is conducted in every sector. The use of blockchain technology may be an excellent solution to ensure reliability, transparency and security, especially for those commodities susceptible to fraud. With regards to fraud the synergic approach of RFID technologies and blockchain is perfect for applications in other contexts related to the agri-food industry. Moreover, the possibilities provided by blockchain systems are certainly an interesting and actual area of future research even if at the moment blockchain seems to suffer from technical limitations and a lack of practical applications.

## Figures and Tables

**Table 1:** Mean (± Standard Deviation) higher distance measurement calculated from both the long-range and the portable antenna for both circular (4 and 3 cm) and nail (2 cm) shaped tag.

Tags Type	Large Antenna		Small Antenna
	Tag Dimensions	Average ±	
Coin shaped	4 cm	450.0 ± 17.0	4.4 ± 0.4
	3 cm	505.1 ± 14.3	18.2 ± 0.2
Nail shaped	2 cm	229.2 ± 16.3	1.5 ± 0.3
